# Comparison of Iron Profile in Patients With and Without Coronary Heart Disease

**DOI:** 10.7759/cureus.15613

**Published:** 2021-06-12

**Authors:** Zain Amar, Abdul Subhan Talpur, Shumaila Zafar, Asadullah Memon, Kefayatullah Nazary, Saliman Esmati, Sara Hashim, Hamza Maqsood, Farukhzad Hafizyar, Besham Kumar

**Affiliations:** 1 Internal Medicine, Isra University, Hyderabad, PAK; 2 Internal Medicine, Liaquat University of Medical and Health Sciences, Jamshoro, PAK; 3 Internal Medicine, Mayo Hospital, Lahore, PAK; 4 Emergency Medicine, Kohsar Hospital, Hyderabad, PAK; 5 Internal Medicine, Kabul University of Medical Sciences, Kabul, AFG; 6 Internal Medicine, Jamaica Hospital Medical Center, New York City, USA; 7 Pathology and Laboratory Medicine, Bolan Medical College, Quetta, PAK; 8 Internal Medicine, Nishtar Medical University, Multan, PAK; 9 Internal Medicine, Ariana Sabet Hospital, Kabul, AFG; 10 Internal Medicine, Jinnah Postgraduate Medical Centre, Karachi, PAK

**Keywords:** iron, ferritin, oxidized low density lipoprotein, serum iron level, coronary artery disease, association

## Abstract

Introduction

Atherosclerosis is considered a major cause of coronary artery disease (CAD). Pathogenesis of atherosclerosis involves the oxidation of low-density lipoprotein (LDL) within the lysosomes of macrophages. Ferritin and iron have pro-oxidant properties, and ferritin is an independent positive determinant of oxidized LDL level. In this study, we will determine the association between ferritin and serum iron levels and CAD.

Methods

This case-control study was conducted in the cardiology unit of a tertiary care hospital in Pakistan from December 2020 to April 2021. After taking informed consent, 400 patients with a confirmed diagnosis of CAD were enrolled. Another set of 400 patients without a history of CAD were included in the control group. A blood sample of 5 ml was drawn and sent to the laboratory to test for ferritin, serum iron, and total iron-binding capacity (TIBC). Ferritin, serum iron, and iron-binding capacity were compared between the case and control groups.

Results

Serum ferritin was significantly higher in patients with CAD compared to patients without CAD (921.21 ± 201.21 ug/L vs. 101.21 ± 92.21 ug/L; p-value: <0.0001). Serum TIBC was significantly lower in patients with CAD compared to patients without CAD (302.12 ± 101.75 umol/L vs. 362.12 ± 82.16 umol/L).

Conclusion

Patients with raised levels of ferritin should consult a physician to manage their ferritin levels since they are at a greater risk of CAD. Treatment ranges from lifestyle changes to pharmacological therapy, thus reducing the overall risk and normalizing the ferritin levels.

## Introduction

Coronary artery disease (CAD) is a common heart condition that involves atherosclerotic plaque formation in the vessel lumen. This impedes blood flow and thus oxygen delivery to the myocardium [1]. Since the last several decades, the rates of cardiovascular disease-related deaths have decreased in many high-income countries. However, in low and middle-income countries, including Pakistan, it has increased and is responsible for 80% of the global disease burden [2]. Atherosclerosis is considered the major cause of CAD [3]. One of the early steps of atherosclerosis is a focal accumulation of lipid-laden foam cells derived from macrophages. Before the formation of foam cells, there is a localized attachment of circulating monocytes to the arterial endothelial cells [4]. Various studies have now proven that the oxidized low-density lipoprotein (ox-LDL)-cholesterol is more important in the formation and progression of atherosclerosis than the native unmodified LDL-cholesterol [5]. Ox-LDL acts via binding scavenger receptors, upregulation of its receptor, growth, and migration of the smooth muscle cells, monocytes/macrophages and fibroblasts, and generation of reactive oxygen species [5].

LDL has recently been shown to be oxidized by iron within the lysosomes of macrophages, and this is a novel potential mechanism for LDL oxidation in atherosclerosis [6]. Other studies have also shown that ferritin and iron have pro-oxidant properties, and ferritin is an independent positive determinant of ox-LDL level [7,8]. The role of iron that is relevant to atherosclerosis and CAD has not yet been properly investigated, particularly in developing countries. In this study, we will determine the association between ferritin and serum iron levels and CAD.

## Materials and methods

This case-control study was conducted in the cardiology unit of a tertiary care hospital in Pakistan from December 2020 to April 2021. After taking informed consent, 400 patients with a previously confirmed diagnosis of CAD and another set of 400 patients without a history of CAD were included in the intervention and control group, respectively. Case and control groups were enrolled from the outpatient department via consecutive convenient non-probability sampling. Ethical approval was obtained from the Institutional Review Board (IRB) of Isra Hospital before the initiation of the study. The study subjects were thoroughly informed about the nature of the study and provision for voluntary withdrawal at any stage of the study while maintaining the confidentiality of the study participants. Patients having chronic inflammatory and infective diseases, and those on iron medication were excluded from the study.

After taking informed consent, demographics and characteristics such as age, gender, smoking history, BMI, hypertension, and diabetes status were noted in a self-structured questionnaire. A blood sample of 5 ml was drawn from both case and control groups via phlebotomy and sent to the laboratory to test for ferritin, serum iron, and total iron-binding capacity (TIBC). The blood sample was collected in the morning to avoid diurnal fluctuation.

Statistical analysis was done using the Statistical Package for the Social Sciences (SPSS) v. 21.0 (IBM Corporation, Armonk, New York, United States). Continuous variables were analyzed using descriptive statistics and presented as mean and SD. Categorical variables were presented as percentages and frequencies. Ferritin, serum iron, and TIBC between the two groups were compared using an independent t-test. A p-value of less than 0.05 meant that there is a difference between the case and control group and the null hypothesis is void.

## Results

There were more males in the study, but the ratio of males to females in both groups was similar. There was no significant difference in other characteristics between groups (Table 1).

**Table 1:** Characteristics of the study participants. CAD: Coronary artery disease; NS: Nonsignificant.

Characteristics	Patients with CAD (n = 400)	Patients without CAD (n = 400)	P-value
Age in years (mean ± SD)	51 ± 11	51 ± 10	NS
Male (%)	251 (62.75%)	249 (62.25%)	NS
Hypertension (%)	312 (78.0%)	309 (77.25%)	NS
Diabetes (%)	152 (38.0%)	158 (39.5%)	NS
Smoking (%)	115 (28.75%)	111 (27.75%)	NS
BMI kg/m^2^	24.12 ± 4.12	24.12 ± 4.12	NS

Serum ferritin was significantly higher in patients with CAD compared to the patients without CAD (921.21 ± 201.21 ug/L vs. 101.21 ± 92.21 ug/L; p-value: <0.0001). Serum TIBC was significantly lower in patients with CAD compared to patients without CAD (Table 2).

**Table 2:** Comparison of iron profile in patients with and without CAD. CAD: Coronary artery disease; NS: Nonsignificant; TIBC: Total iron-binding capacity. ug: Microgram; umol: Micromole.

Characteristics (mean ± SD)	Patients with CAD (n = 400)	Patients without CAD (n = 400)	P-value
Ferritin (ug/L)	921.21 ± 201.21	101.21 ± 92.21	<0.0001
Serum Iron (umol/L)	74.12 ± 22.54	77.25 ± 24.73	NS
TIBC (umol/L)	302.12 ± 101.75	362.12 ± 82.16	<0.0001

## Discussion

In our study, patients with CAD reported elevated levels of serum ferritin compared to those who did not have CAD. Several other studies have shown a similar positive correlation, suggesting that with increased stores of iron within the body, the risk of CAD is increased from 1.6 to 6.7 times [9-12]. On the other hand, a population-based cohort study by Reyes et al. demonstrated that the higher risk of CAD is not related to the serum ferritin levels [13]. This discrepancy is the result of a study conducted by Reyes et al. could be explained by the fact that the area in which this study was conducted has generally reported a lower prevalence of cardiovascular diseases [13]. Another important finding of our study suggested reduced TIBC in CAD patients compared to those without CAD. A case-control study conducted by Pourmoghaddas et al. also anchored the findings of our study [14]. Pourmoghaddas' study was conducted in Iran which showed that high iron store, as assessed by serum ferritin, was associated with the increased risk of CAD [14].

There is sufficient literature available globally explaining the positive correlation between ferritin and CAD. This could be explained by the well-known fact that increased oxidation of LDL potentially leads to atherosclerosis. Since iron is readily oxidized and reduced, it is easier to produce ox-LDL and oxygen-free radicals. Both these agents can cause damage at a vascular level and lead to atherosclerosis [15]. Despite these biological mechanisms being suggested as the potential causes, the pathology still requires further clarification [16]. The raised serum ferritin is due to multiple causes, including conditions of inflammation and infection. Moreover, hepatic and renal diseases and malignancies are also some of the most frequent factors that elevate ferritin levels [17]. This denotes that these factors which could cause increased ferritin level may also increase the risk of CAD. Therefore, patients with any of the mentioned risk factors should have their iron levels screened at regular intervals. Proper screening and timely check-ups would help in lowering the risks of CAD. 

To the best of our knowledge, this is the first study in the local population that studies the relation between CAD and ferritin. However, since this study was conducted in a single institute, care should be taken while inferring the result to a greater sample size. Further large-scale trials are needed that study the direct correlation between ox-LDL and ferritin level to further confirm the findings of our study.

## Conclusions

In our study, patients with CAD had higher ferritin levels, which suggests a potential role of ferritin in increasing the risk of cardiovascular disease. Cardiovascular diseases are associated with higher mortality rates; atherosclerosis is one of the causes of the development of heart-related diseases. Therefore, patients with raised levels should consult a physician to control their ferritin levels, since they are at a greater risk of cardiac disorders. Treatments could range from lifestyle changes to pharmacological therapy, thus reducing the overall risk and normalizing the ferritin levels.
